# Transcriptome responses of *Lactobacillus acetotolerans* F28 to a short and long term ethanol stress

**DOI:** 10.1038/s41598-017-02975-8

**Published:** 2017-06-01

**Authors:** Xiaopan Yang, Kunling Teng, Jie Zhang, Fangfang Wang, Tong Zhang, Guomin Ai, Peijie Han, Fengyan Bai, Jin Zhong

**Affiliations:** 1 State Key Laboratory of Microbial Resources, Institute of Microbiology, Chinese Academy of Sciences, Beijing, 100101 People’s Republic of China; 2 University of Chinese Academy of Sciences, Beijing, 100101 People’s Republic of China; 3 State Key Laboratory of Mycology, Institute of Microbiology, Chinese Academy of Sciences, Beijing, 100101 People’s Republic of China

## Abstract

*Lactobacillus acetotolerans* is a major microbe contributing to the Chinese liquor fermentation with unknown function. It can be grown well in a high concentration of ethanol. RNA sequencing (RNA-seq) was performed on *L. acetotolerans* F28 growing in 12% ethanol to determine important genetic mechanisms for both a short and long term adaption to this environment. A genome-wide transcriptional analysis revealed that the most important genetic elements for *L. acetotolerans* F28 grown in ethanol are related to high levels of stress response and fatty acid biosynthesis, and a reduction of amino acid transport and metabolism after both a short and long time stress. The fatty acid methyl ester analyses showed that most fatty acids were increased in *L. acetotolerans* F28 after exposure to ethanol while the unsaturated fatty acid octadecenoic acid (C18:1) was significantly increased. The increasing unsaturated fatty acid biosynthesis in *L. acetotolerans* F28 might enhance cell membrane fluidity and protect the cells against high concentration of ethanol. Overall, the transcriptome and functional analysis indicated that the elevated stress response and fatty acid biosynthesis, and the decrease of amino acid transport and metabolism might play important roles for *L. acetotolerans* F28 to adapt to environmental ethanol.

## Introduction

*Lactobacillus* is the largest genus of lactic acid bacteria and is widely used in various foods and beverages fermentation including yoghurt, sausage, wine and liquor^1–4^. During the fermentation processes, *Lactobacillus* is exposed to several stresses, such as high content of acids or alcohol, low or high temperature, growth-inhibitory compounds and so on. They are required to survive and grow under specific stress environmental conditions^5^. Chinese liquor is a grain fermented alcoholic beverage widely consumed in China. In the liquor fermentation process, the fermented grains are the specific fermentation matrix for microorganisms. Recent studies about bacterial communities and diversities in Chinese liquor fermentation processes using nonculture-based methods showed that lactobacilli are major microbes contributing to the liquor fermentation^4, 6^. *Lactobacillus acetotolerans* is the dominant specie, and its percentage among the bacteria is more than 80% from day 9 to 40 during the liquor fermentation in which the ethanol content can reach more than 12% (v/v)^6^. Besides, *L. acetotolerans* had positive relationships with most chemical components that contribute to the quality and flavor of liquor. As a result, *L. acetotolerans* is thought to be very important for the production of liquor.

*L. acetotolerans* is also found in Japanese sake and spoiled beer^7, 8^. The ethanol content of Japanese sake is more than 15% (v/v) in fermentation process, which is one of the highest ethanol content among the fermented beverages. Most lactic acid bacteria have no tolerance to this level of ethanol and stop growing in an environment containing more than 12% (v/v) ethanol^8, 9^. A *L. acetotolerans* strain from Japanese sake has been reported to have high ethanol-tolerance and its whole genome has been sequenced^8^. However, there is limited knowledge about how *L. acetotolerans* respond to the high content of ethanol in the environment, and its molecular mechanism of ethanol-tolerance have not been fully understood.

*Oenococcus oeni* is the dominant bacteria which perform malolactic fermentation during wine making. Thus ethanol tolerance is very important for *O. oeni*. The transcriptomic and proteomic analysis of *O. oeni* adaptation to wine stress conditions has been reported^10^. A small heat shock protein named Lo18 was also identified as a major stress protein to prevent protein aggregation and stabilizing the cytoplasmic membrane^11^. As *Lactobacillus* are major microbes contributing to the liquor fermentation, understanding the mechanisms of ethanol tolerance of *Lactobacillus* is of great importance. Several studies have examined the proteins or genes in response to environmental ethanol. Global transcriptome profiling using DNA microarrays in *Lactobacillus plantarum* treated by 8% ethanol demonstrated that ethanol exposure led to induced expression of genes involved in citrate metabolism, cell envelope architecture and canonical stress response pathways regulated by CtsR^12^. Ethanol responsive proteins were also identified by the proteomic approach in *Lactobacillus buchneri* treated by 10% ethanol. The changes suggested that *Lactobacillus buchneri* respond to ethanol by proteins and fatty acids degradation and increased production of specific enzymes and molecular chaperones^13^. However, it is unclear if the above genetic pathways are important for the growth of *L. acetotolerans* under ethanol stress.

RNA sequencing (RNA-seq) is a powerful technology to identify the transcriptional profiles of specific bacteria even the one whose genome has not been sequenced^14^. Gene transcription and expression levels, as well as molecular mechanisms involved in bacteria growth under different ecological conditions can be obtained. *L. acetotolerans* strain F28 was isolated from the fermented grains of Fen liquor which is one of the grain fermented alcoholic beverages consumed in China on a medium containing 10% ethanol. Here we described the global transcriptomic changes of *L. acetotolerans* F28 treated with a short and long term 12% concentration of ethanol through RNA-seq technology. We aimed to determine the general adaptive and protective responses of *L. acetotolerans* F28 during growth in the presence of ethanol. Gene variations associated with the adaption of *L. acetotolerans* to ethanol in both a short and a long time stage were revealed.

## Results

### Identification and growth of *L. acetotolerans* F28

**Figure 1:**
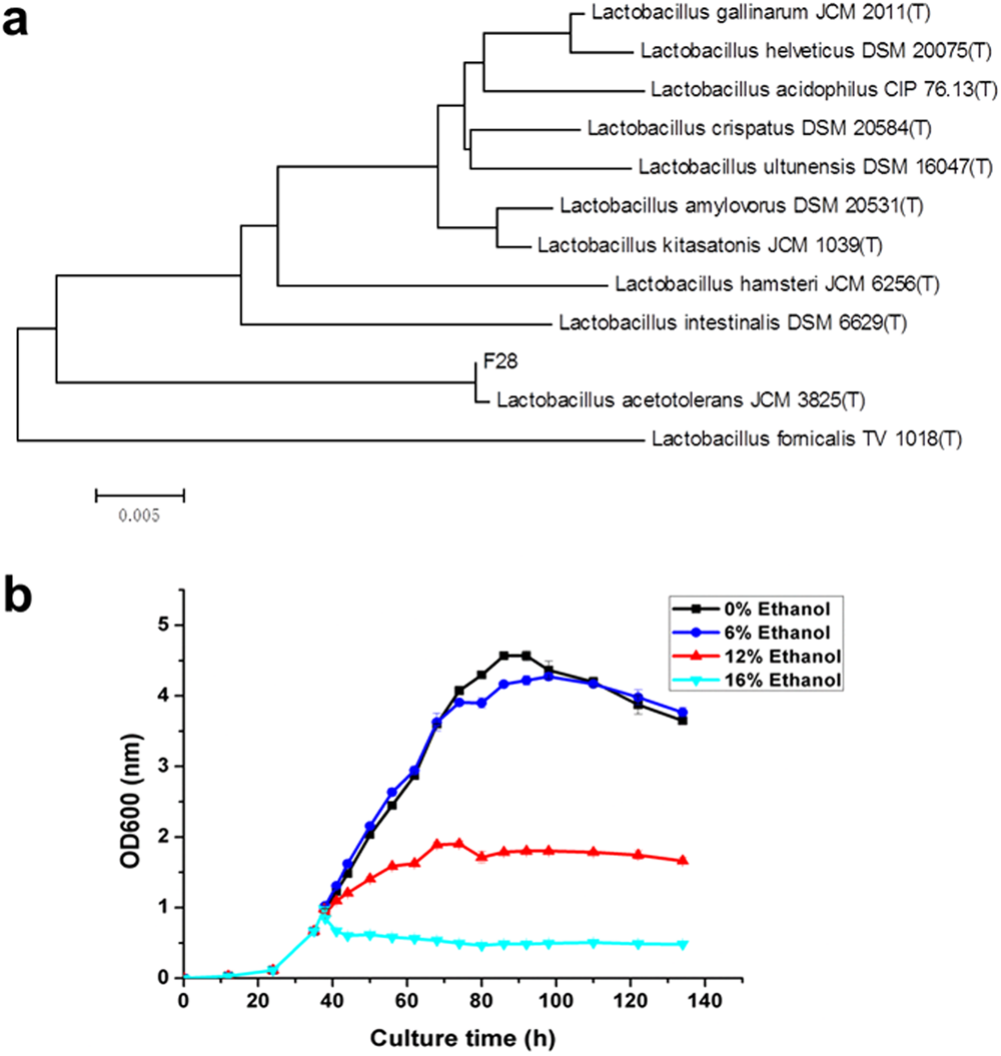
Identification and growth curve of *L. acetotolerans* F28. (**a**) Phylogenetic tree based on 16 S rRNA gene sequence comparisons showing the position of strain F28 and related species of the genus *Lactobacillus*. (**b**) Growth curve of in MRS5 medium in the presence of various concentrations (v/v) of ethanol. Ethanol was added when OD_600_ = 1.0.

A strain named F28 was isolated from the fermented grains of Fen liquor on a MRS5 (MRS broth medium with pH value is 5) medium containing 10% ethanol for 6 days. The 16 S rRNA sequence of F28 showed 99.93% similarity to that of *L. acetotolerans* JCM 2825 (Fig. 1a). Thus, strain F28 was supposed to be a *L. acetotolerans* strain. *L. acetotolerans* F28 showed a comparable long growth term in which the cells maintained the exponential phase from the 24^th^ to 84^th^ hours, and then entered into the stationary phase (Fig. 1b).

When ethanol was added to the culture medium (OD_600_ = 1.0) at a final concentration of 6% (v/v), the growth of *L. acetotolerans* F28 showed no significant difference compared to that without ethanol. Cell growth was suppressed but still kept well by 12% (v/v) ethanol. When ethanol was added to a final concentration of 16% (v/v), *L. acetotolerans* F28 stopped growing and the optical density was decreased rapidly (Fig. 1b). These results demonstrated that *L. acetotolerans* F28 was able to resist a comparable high concentration of ethanol ( > 12%).

### General genetic expression of *L. acetotolerans* F28 responding to ethanol

**Figure 2:**
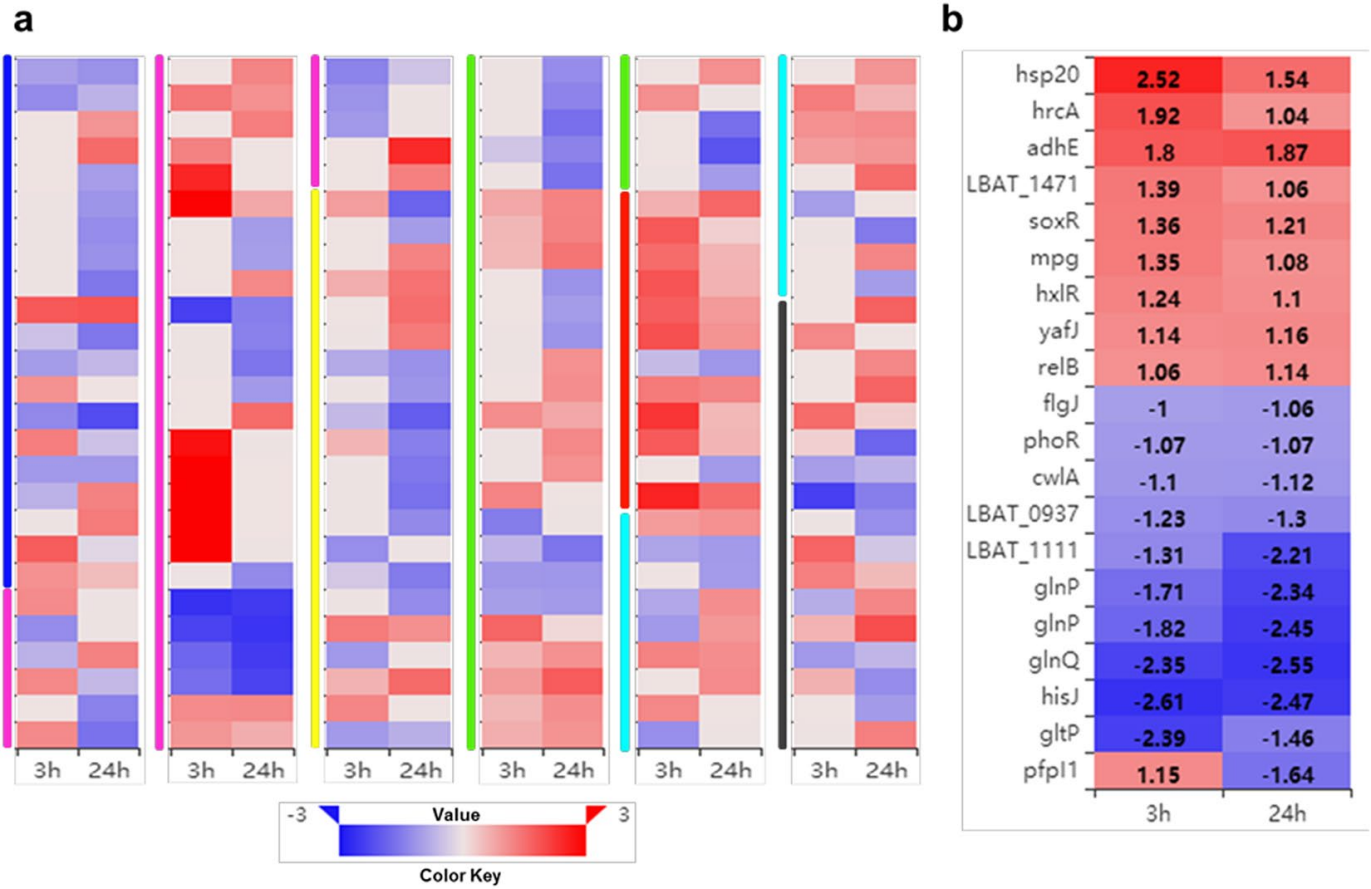
Heatmap of log_2_ fold-change value of differently expression genes in *L. acetotolerans* F28 during ethanol treatment. (**a**) Classification of significantly differentially expression genes. Color code for different classes: blue: carbohydrate transport and metabolism, pink: nitrogen transport and metabolism, yellow: replication, transcription, translation and repair, green: cell division and envelope biogenesis, red: stress response, cyan: defense mechanisms, black: others. The class of hypothetical protein was not shown in this figure. (**b**) Core genes of significantly differentially expression at both time points. 3 h: 3 hours after exposing to ethanol; 24 h: 24 hours after exposing to ethanol.

The transcriptome of *L. acetotolerans* F28 after a short 3 hours and longer 24 hours incubation in MRS medium containing 12% (v/v) ethanol were identified using RNA-seq. Illumina paired-end sequencing of 12 samples yielded a total of 105,030,954 clean reads. 67.8% to 71.3% reads of each sample was mapped to the annotated *L. acetotolerans* genome NBRC 13120. Significantly differentially expressed genes at 3 and 24 hours were identified (q ≤ 0.05, log_2_ fold-change ≥ 1) by comparing the gene expression profiles of treated cells to that of control cells. 81 genes were significantly differentially expressed after treated with ethanol for 3 hours, of which 54 were up-regulated and 27 were down-regulated (Supplementary Table S1). After 24 hours treatment with ethanol, the number of significantly differentially expression genes was up to 130 in which 66 were up-regulated and 64 were down-regulated (Supplementary Table S2). Their functions are related to carbohydrate transport and metabolism, nitrogen transport and metabolism, cell division and envelope biogenesis, stress response, defense mechanisms, replication, transcription, translation, repair, coenzyme transport and metabolism, transcriptional regulation and some unknown function genes. They were classified into eight categories and their expressions were shown in Fig. 2A. 20 core genes were significantly differentially expressed at both 3 and 24 hours treatment by ethanol (Fig. 2b). 9 core genes were significantly up-regulated, and they are mainly stress responsive and defense related genes (*hsp20*, *hrcA*, *soxR*, *relB*), a aldehyde-alcohol dehydrogenase *adhE*, a nucleoside triphosphate hydrolase, a 3-methyladenine DNA glycosylase *mpg*, a transcriptional regulator *hxlR* and a glutamine amidotransferase yafJ. 11 core genes were significantly down-regulated, and they are mainly amino acid transport related genes including *hisJ*, *glnQ*, *glnP* and *gltP*, a NAD (FAD)-dependent dehydrogenase *cwlA*, a two-component sensor kinase encoding gene *phoR* and a N-acetylmuramidase encoding gene *flgJ*.

**Figure 3:**
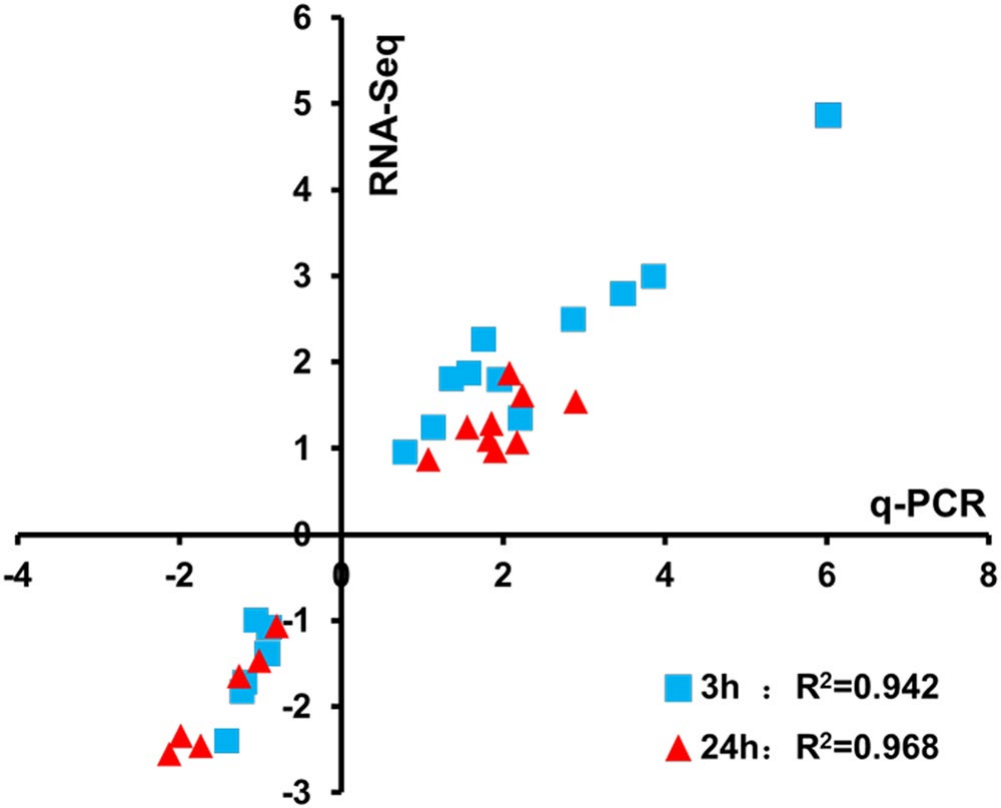
Correlation between RNA-Seq and real-time qRT-PCR results for RNA-Seq data verification. The gene expression ratios of both RNA-Seq data and qPCR data were log transformed in base 2 (log_2_ < Ethanol_treatment/Control > ), and RNA-Seq log_2_ ratio values were plotted against the qRT-PCR log_2_ values. Twenty four genes tested are *glnP* (LBAT_0325), *glnP* (LBAT_0326), *glnQ*, *mpg*, *gltP*, *dck*, *adhE*, *groEL*, *pyrR* (LBAT_1269), *uraA* (LBAT_1270), *whiA*, *ldhA*, LBAT_0175, *acm*, *dnaK*, *hxlR*, LBAT_0555, *clpE*, LBAT_0514, *hsp20*, *sdhA* (LBAT_0078), *clpB*, *pyrC* and *carB* (LBAT_0598).

Twenty four differentially expressed genes representing different functional categories were selected for real-time quantitative PCR (qPCR) for confirmation (Fig. 3). Correlation coefficient values of R^2^ = 0.946 and 0.968 were obtained for comparisons between RNA-Seq and qPCR for the time points of 3 hours and 24 hours, respectively. The result indicated that RNA-Seq data were of good quality.

### Effects of ethanol on nitrogen transport and metabolism

**Figure 4:**
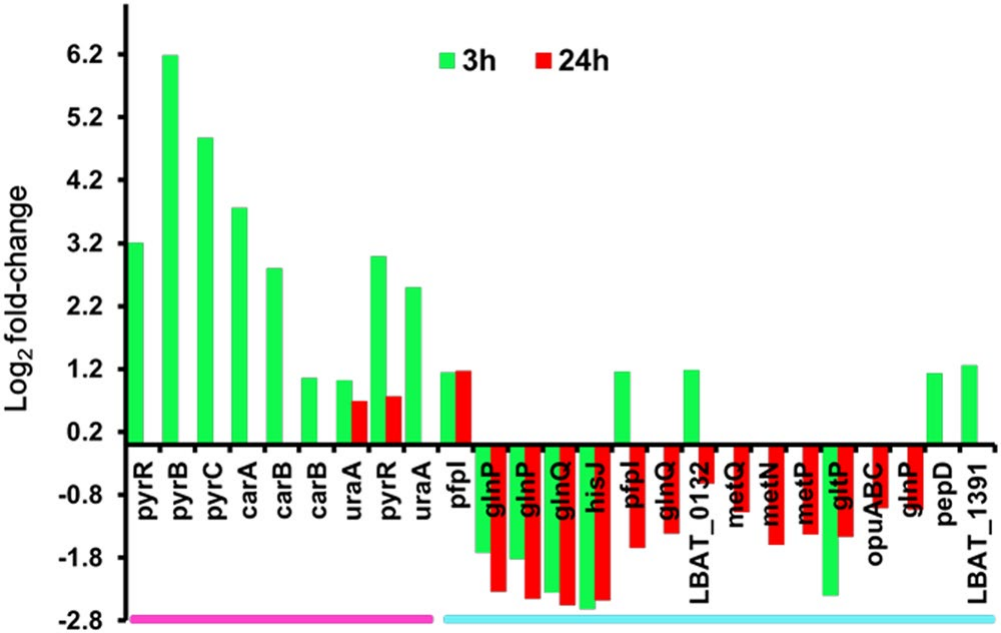
Log_2_ fold-change value of genes related to nitrogen transport and metabolism induced by 12%(v/v) ethanol. Color code: pink: nucleotide transport and metabolism, cyan: amino acid transport and metabolism. 3 h: 3 hours after exposing to ethanol, 24 h: 24 hours after exposing to ethanol.

Ethanol stress could significantly affect the expression of *L. acetotolerans* F28 genes associated with nitrogen transport and metabolism, especially nucleotides and amino acids transport and metabolism (Fig. 4). After treatment with ethanol for 3 hours, genes related to uracil nucleotides synthesis were largely up-regulated. The gene cluster involving in uracil nucleotides de novo synthesis including *pyrB* (aspartate carbamoyltransferase catalytic subunit), *pyrC* (dihydroorotase), *carA* (carbamoyl phosphate synthase small subunit), *carB* (carbamoyl phosphate synthase large subunit) were hardly expressed in control while up-regulated up to 72-fold in samples treated with ethanol for 3 hours. Similarly, genes which worked in salvage pathways including *pyrR* (uracil phosphoribosyltransferase) and *uraA* (uric acid permease) were also up-regulated. However, when treated for 24 hours, the expressions of above genes did not show significant differences compared to control. These results demonstrated that the synthesis of uracil nucleotides might be stimulated by ethanol in a short time and went back to the normal level with a longer adaption time.

Genes involving in amino acids transport and metabolism were also significantly differentially expressed during ethanol stimulation. After treatment with ethanol for 3 hours, 10 related genes were differentially expressed. As Fig. 4 shown that *pepD* (dipeptidase) and two *pfpI* (glutamine amidotransferase) genes which were involved in peptides degradation were up-regulated by 1.18- to 1.22-fold compared to that of control. These results demonstrated that ethanol promote peptide degradation in cells in 3 hours. At the same time, genes that may participate in polar amino acid (especially glutamine) transport including *gltP* (sodium/dicarboxylate symporter), *hisJ* (amino acid ABC transporter substrate binding component), *glnQ* (amino acid ABC transporter ATP-binding component) and two *glnP* (polar amino acid ABC transporter permease component) genes were down-regulated by 1.1- to 5.1-fold compared to control. Till 24 hours, these genes were maintained the down-regulated expressions. These results indicated that high concentration of ethanol might lower the transport and biosynthesis of amino acid in *L. acetotolerans* F28. Except these five genes, other genes related to polar amino acid import including *opuABC* (glycine/betaine ABC transporter permease), another *glnP* (glutamine ABC transporter permease, LBAT_1257), *metQ* (ABC transporter ATP-binding component), *metN* (methionine ABC transporter ATP-binding component), *metP* (methionine ABC transporter permease), and another *glnQ* (amino acid ABC transporter ATP-binding component, LBAT_0131) were also down-regulated in samples treated by ethanol for 24 hours. Although two genes (LBAT_0132 and LBAT_1391) related to amino acid transport were slightly up-regulated at 3 hours, these results demonstrated that the amino acid import was severely impaired when *L. acetotolerans* F28 was grown in ethanol either in a short response or a longer response.

### Stress and defense response in *L. acetotolerans* F28 during growth in ethanol

After a short stimulating (3 hours), many genes involving in stress response pathway were rapidly increased by 1.3- to 4.7- fold (Table 1). Expression of *hsp20* (heat shock protein) was enhanced to 5.7 fold. A gene *soxR* encoding MerR family transcriptional regulator which can promote transcription of various stress regulons was up-regulated to 2.56 fold. Transcription of *hrcA* and genes which were predicted to be regulated or partially controlled by HrcA including *dnaK* (coding for a heat shock protein), *dnaJ* (coding for a chaperone protein), *clpE* (coding for Clp protease), chaperone encoding genes *groES*, *groEL*, and *grpE* were also up-regulated by 1.91- to 3.81- fold. The chaperone machineries GroES-GroEL and DnaK-DnaJ-GrpE are involved in diverse cell processes such as protein folding and degradation, assembly of protein complexes, and transport of proteins across membranes^5^. These results suggested that *L. acetotolerans* F28 might deal with ethanol stress by increasing expression of chaperonins and proteases to prevent misfolding and aggregation of protein molecules.

**Table 1:** The fold change value of genes, grouped into stress response and defense mechanism pathway after exposing to 12% (v/v) ethanol for 3 hours or 24 hours.

Fuctional group	Gene	Protein encoded	3 hous	24 hours
Stress response	*hsp20*	Heat shock protein	5.73	2.91
	*clpC*	ATP-dependent Clp protease ClpC	**—**	0.48
	*groES*	Chaperone GroES	3.43	1.50
	*groEL*	Chaperone GroEL	4.81	1.47
	*soxR*	MerR family transcriptional regulator	2.56	2.32
	*lexA*	LexA repressor	0.67	0.46
	*hrcA*	Heat-inducible transcription repressor	3.77	2.06
	*grpE*	Heat shock protein GrpE	3.32	1.94
	*dnaK*	Molecular chaperone DnaK	3.66	1.55
	*dnaJ*	Chaperone protein DnaJ	2.91	1.52
	*clpE*	Clp protease ClpE	3.49	1.21
	*clpB*	ATP-dependent Clp protease	1.58	3.06
Defense mechanisms	*hxlR*	HxlR transcriptional regulator	2.36	2.15
	LBAT_0175	XRE-family transcriptional regulator	0.47	1.97
	LBAT_0176	XRE-family transcriptional regulator	0.55	2.13
	*yadH*	ABC transporter permease component	**—**	2.28
	LBAT_1370	MBL fold metallo-hydrolase	**—**	0.35
	LBAT_1464	TetR family transcriptional regulator	**—**	2.9
	*relB*	RelB suprefamily	1.88	2.03
	*trxA*	Thiol reductase thioredoxin	**—**	2.03

—: no significant difference (q value > 0.05).

At 24 hours, *soxR*, *hrcA* and *hsp20* maintained the significantly up-regulated states, and other related genes up-regulated at 3 hours also showed up-regulated to 1–2 fold. An ATP-dependent Clp protease ATP-binding subunit encoded gene *clpB* was up-regulated by 2.06 fold. *clpB* is predicted to be a chaperon for protein disaggregation facilitate de novo protein folding in stressed conditions. This means that *L. acetotolerans* F28 adapted to ethanol stress by up-regulating a series of stress response genes to protect itself away from being injured. At this time, more genes especially that encoding transcriptional regulator or ABC transporter related to cell defense were also up-regulated (Table 1).

### Effects of ethanol on cell envelope composition, cell division, and morphology

**Figure 5:**
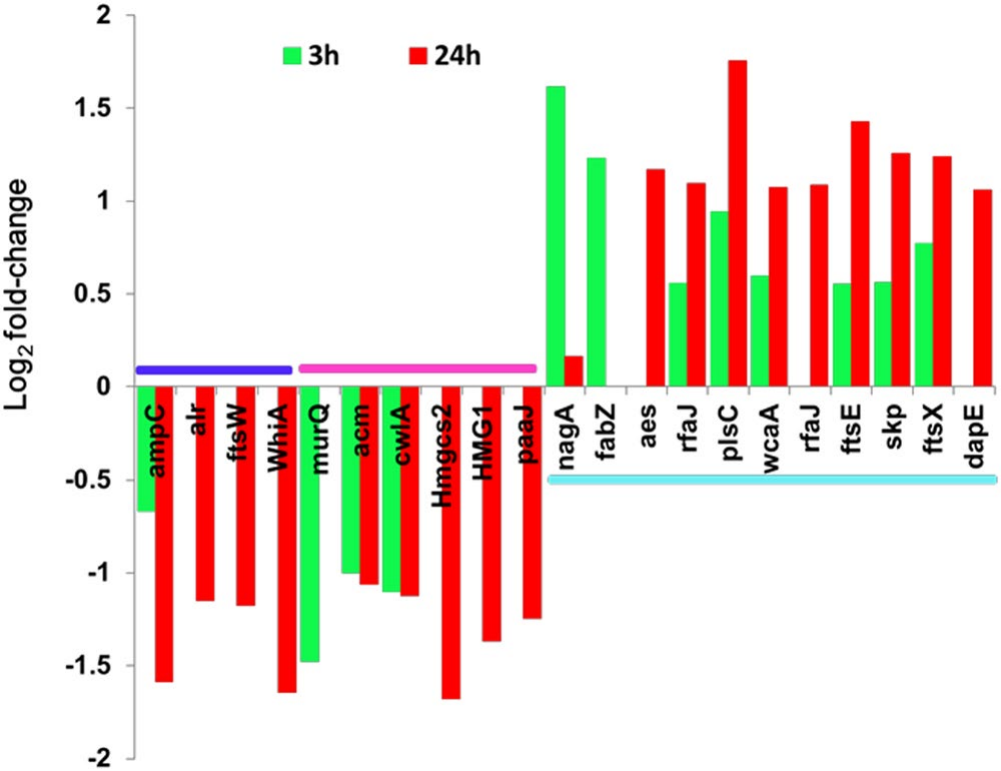
Log_2_ fold-change value of genes related to cell division and envelope biogenesis induced by 12%(v/v) ethanol. Color code: blue: cell division, pink: cell wall degradation, cyan: fatty acids or lipid synthesis. 3 h: 3 hours after exposing to ethanol, 24 h: 24 hours after exposing to ethanol.

According to RNA-seq analysis, the cell wall components related genes of *L. acetotolerans* were influenced by ethanol either in 3 hours or 24 hours. As Fig. 5 shown that genes required for cell wall degradation including *acm* (N-acetylmuramidase), *cwlA* (N-acetylmuramoyl-L-alanine amidase) and *murQ* were decreased by 1- to 1.7-fold after ethanol treated for 3 hours. At 24 hours, except *acm* and *cwlA*, more genes in a gene cluster including *hmgcs2*, *hmg1*, *paaJ* that could convert long-chain fatty acids to coenzyme A and mevalonate were down-regulated 1.0- to 2.2-fold. These results indicated that the cell wall degradation was decreased after ethanol treatment.

Genes needed for fatty-acids or glycerophospholipid biosynthesis including *nagA, fabZ* and *plsC* were induced by 1.1- or 2.0-fold at 3 hours, indicating that the cell wall remodeling was enhanced in this time. At 24 hours, more genes participated in fatty acids or lipid biosynthesis were enhanced. For example glycosyltransferase *rfaJ* and *wcaA*, ABC transporter coding genes *ftsE* and *ftsX*, *aes* (LBAT_0627 and LBAT_0628) which were supposed to degrade lipid to fatty acids were all up-regulated (Fig. 5). These results demonstrated that the fatty acids started to be accumulated after ethanol treatment. A long time stimulation of ethanol might promote more fatty acids to be produced to protect the cells.

**Figure 6:**
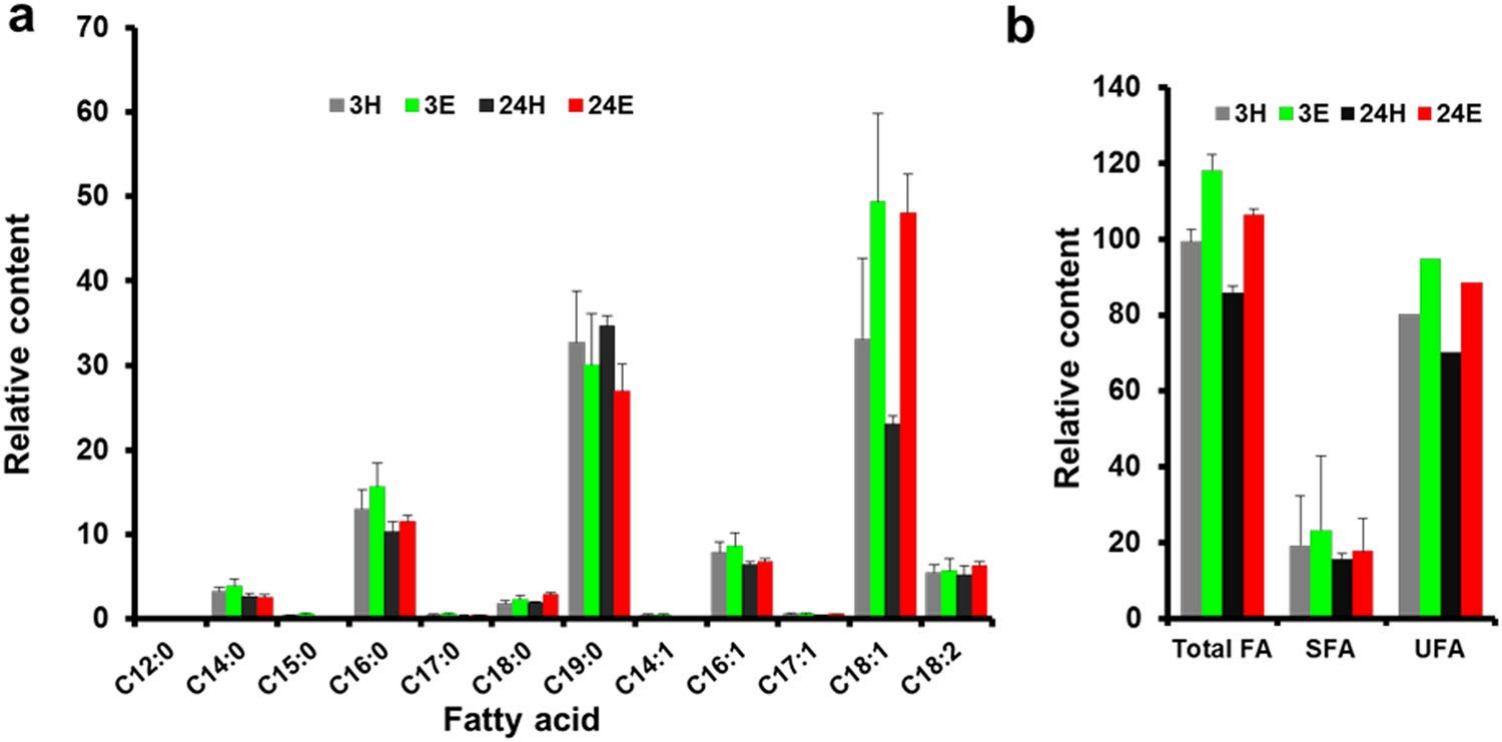
Composition and contents of fatty acids in *L. acetotolerans* F28 grown under stress of 12% (v/v) ethanol. (**a**) Fatty acids composition of *L. acetotolerans* F28 grown under stress of 12% ethanol. (**b**) Fatty acid contents of *L. acetotolerans* F28 grown under stress of 12% ethanol. SFA: saturated fatty acid, UFA: unsaturated fatty acid. 3 H: samples of 3 hours after 12% water treatment (control), 24 H:samples of 24 hours after 12% water treatment(control), 3E:samples of 3 hours after 12% ethanol treatment, 24E:samples of 24 hours after 12% ethanol treatment. Each sample was tested in triplicate.

Our fatty acid methyl ester (FAME) analyses showed that among the 12 kinds of fatty acids including saturated fatty acid and unsaturated fatty acid, cyclopropaneoctanoic acid (C19:0) and octadecenoic acid (C18:1) were main FAs which occupied 60–70 percent of total FAs in *Lactobacillus acetotolerans* F28. The contents of fatty acids in *Lactobacillus acetotolerans* F28 treated with 12% (v/v) ethanol for 3 hours and 24 hours were analyzed. During ethanol treatment, most fatty acids were increased, especially octadecenoic acid (C18:1) that had a significant increase to nearly 2 fold at 24 hours (p = 0.018) (Fig. 6). As a result, total fatty acids including both saturated fatty acids and unsaturated fatty acids were all increased after either 3 hours or 24 hours ethanol treatment (Fig. 6). The increase of unsaturated fatty acids octadecenoic acid (C18:1) content indicated an enhanced cell membrane fluidity, suggesting that the adaptation of cells to ethanol caused by a more fluid membrane. And this conclusion was consistent with our above RNA-seq results.

**Figure 7:**
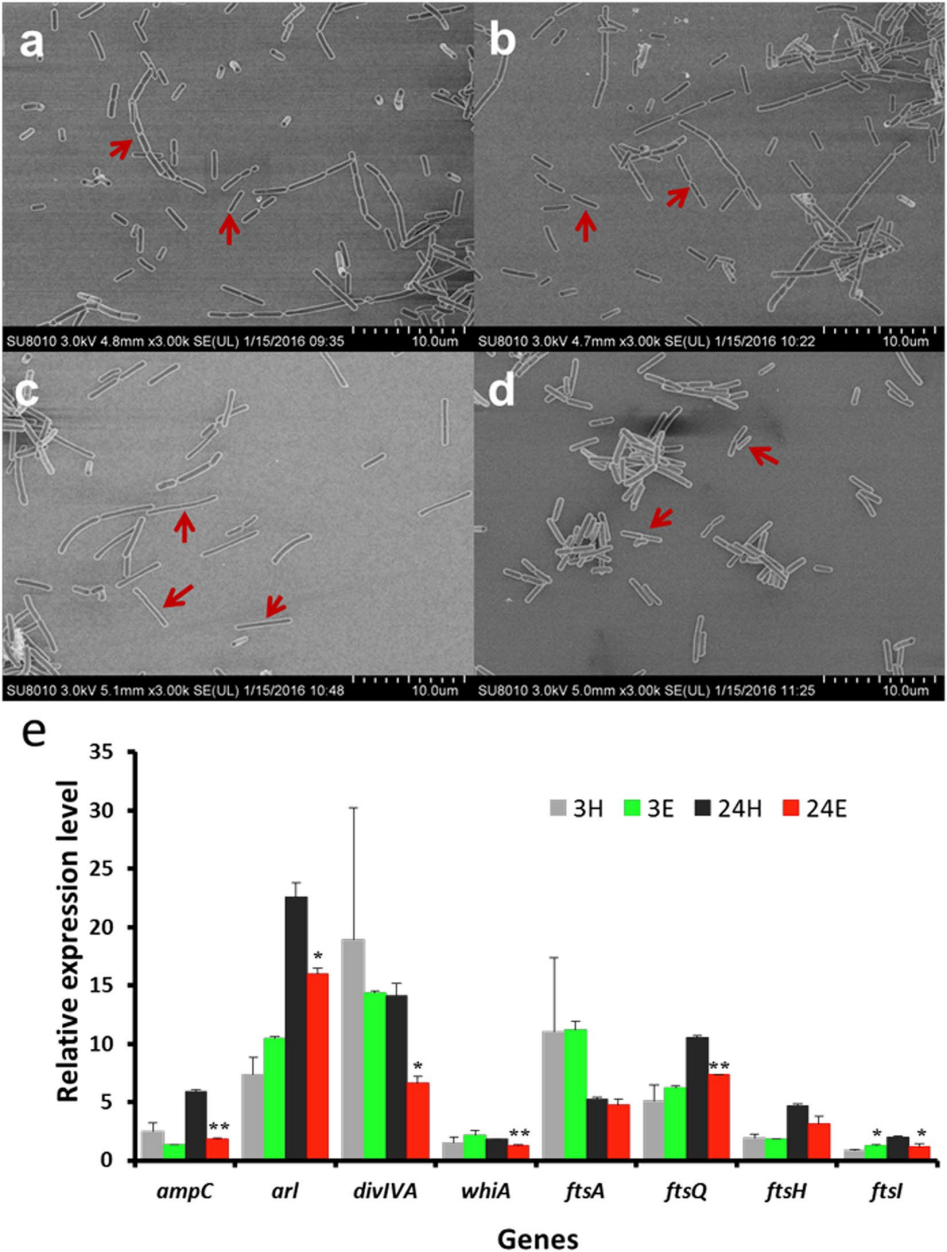
SEM analysis of *L. acetotolerans* F28 grown under the stress of 12% (v/v) ethanol. (**a**) Cell morphology of *L. acetotolerans* F28 after ethanol treatment for 3 hours. (**b**) Cell morphology of *L. acetotolerans* F28 after water treatment for 3 hours (control). (**c**) Cell morphology of *L. acetotolerans* F28 after ethanol treatment for 24 hours. (**d**) Cell morphology of *L. acetotolerans* F28 after water treatment for 24 hours (control). (**e**) The relative expression level of genes related to cell division in *L. acetotolerans* F28 by qRT-PCR. 3 H: samples of 3 hours after 12% water treatment (control), 24 H:samples of 24 hours after 12% water treatment (control), 3E:samples of 3 hours after 12% ethanol treatment, 24E:samples of 24 hours after 12% ethanol treatment. Each sample was tested in triplicate. *Values and standard deviation are calculated from at least three independent experiments performed in triplicate. Significance was determined by Student’s t test. *indicated the significance of p < 0.05 and **indicated the significance of p < 0.01.

The expression levels of genes related to cell division were almost not affected after a short stimulation by ethanol (3 hours). Through a long time adaption (24 hours), genes coding for *alr* (alanine racemase), *ftsW* protein (cell division related membrane protein), *ampC* (penicillin-binding protein) and *whiA* (sporulation regulator) were down-regulated by 1.2, 1.3, 2.0 and 2.1 fold, respectively (Fig. 5). There are several reports showing that knockout either of these genes would lead to an increase in cell length^15–18^. To verify the effects of ethanol treatment on cell morphology, microscopic analyses of *L. acetotolerans* F28 treated with ethanol for 3 hours and 24 hours were performed using Scanning Electron Microscope (SEM). *L. acetotolerans* F28 showed similar morphology treated with or without ethanol for 3 hours. While treated with ethanol for 24 hours, *L. acetotolerans* F28 showed a much longer cell length than that treated with water (Fig. 7). This result indicated that the cell length of *L. acetotolerans* F28 might be increased under ethanol stress for a long time adaption. Further, the expression levels of some cell division related genes in *L. acetotolerans* F28 were detected by qRT-PCR to see whether the increase of cell length was caused by cell division. As shown in Fig. 7e that the expression of genes coding for cell division related proteins, including *ampC* (penicillin-binding protein), *alr* (alanine racemase), *divIVA* (cell division initiation protein), *whiA* (sporulation regulator), *ftsA* (cell division protein), *ftsQ* (cell division related protein), *ftsH* (ATP-dependent metalloprotease) and *ftsI* (cell division protein, penicillin-binding protein) did not show obvious difference in *L. acetotolerans* F28 after treated with ethanol for 3 hours. However, after treated with ethanol for 24 hours, most of the above genes showed decreased expression levels by 0.43 to 2.17 fold, except *ftsA* with no big difference. These results suggested that the increased cell length of *L. acetotolerans* F28 under ethanol adaption for a long time might be caused by the inhibition of cell division.

## Discussion

Some *Lactobacillus* species are able to grow and survive under suboptimal conditions during food and beverage fermentations. Fen liquor is a popular grain fermented beverage in China. During the liquor fermentation process, the fermented grains are the specific matrix providing nutritional substances for microorganisms to produce ethanol, microbial metabolites and volatile compounds^4^. The concentration of ethanol in fermented grains can reach up to 12% in this process. *L. acetotolerans* was found to become the dominant specie from the 13^th^ days during the fermentation process using an uncultured sequencing method. This was also supported by other previous report about bacterial communities in Chinese liquor^4, 6, 19, 20^. Although *L. acetotolerans* was also reported to be a kind of beer-spoilage bacteria, it seems to play an important role in liquor production. *L. acetotolerans* strains from spoilage beer are hard to be cultured in routine medium because of its viable putative non-culturable (VPNC) state in the beer^7^. A global transcriptomic changes between the VPNC and normal state of *L. acetotolerans* isolated from spoilage beer was also assayed^21^. The cultural clone counting of *L. acetotolerans* in fermented grains during the fermentation process showed that *L. acetotolerans* bacteria started to grow from 13^th^ days and grow greatly faster from 26^th^ to the end of fermentation. As a result, we supposed that *L. acetotolerans* might grow well instead of being in a VPNC state and played a key role in grains fermentation in liquor production. *L. acetotolerans* was grown well in the late state of grains fermentation means that they are more adapted to ethanol. We have also shown that *L. acetotolerans* F28 was able to survive and grow well in 12% ethanol in MRS5. Understanding the global gene expression profiling of *L. acetotolerans* F28 under ethanol stress may provide valuable insights into its genetic response and adaption to ethanol. To our knowledge, there is no report on genome-wide expression profiling of *L. acetotolerans* under short and long term ethanol stress.

In this study, RNA-seq was performed to determine global gene expression profiling in *L. acetotolerans* F28 to identify the genetic responses of *L. acetotolerans* to ethanol in a short and long time. The significantly up-regulated core genes in *L. acetotolerans* F28 treated by ethanol for both 3 hrs and 24 hrs were mainly stress response and defense related genes. Class I stress response transcriptional regulator encoding gene *hrcA* was up-regulated by more than 1 fold at both time points, indicating that *hrcA* might contribute to *L. acetotolerans* F28 protection to ethanol. Previous report about *Lactobacillus plantarum* showed that *hrcA* was up-regulated at 30 min but not 24 hours after treated by 8% ethanol, and Class I stress response transcriptional regulator CstR was involved in cross protection to ethanol^12^. CstR was also be found correlated to ethanol adaption in *Oenococcus oeni* ^22^. While in *L. acetotolerans* F28, *cstR* gene did not show significantly differential expression compared to control at both time point. The genes which might be regulated or partially controlled by HrcA including *dnaK*, *dnaJ*, *groES*, *groEL*, and *grpE* were up-regulated by 1.91- to 3.81- fold after 3 hours ethanol exposure and 1 to 2 fold after 24 hours ethanol exposure. This was similar to the responses of *L. plantarum* and *O. oeni* that *groES*, *groEL*, and *grpE* were up-regulated at all the time points^10, 12^. Other genes related to environmental stresses were also up-regulated during growth in ethanol. A heat shock protein encoding gene *hsp20* was up-regulated at both time points while in *O. oeni, hsp20* was decreased after exposure to high concentration of ethanol^10, 23^. It is reported that the expression level of *hsp20* was only increased in mild ethanol stress (8%) but not in 12% ethanol in *O. oeni*. Our study found that in *L. acetotolerans* F28, *hsp20* was up-regulated under the stress of 12% ethanol both in short and long time treatment. ATP-dependent Clp protease encoding gene *clpE* was also up-regulated in *L. acetotolerans* F28 in both a short and long time ethanol stress similar to that in *L. plantarum* ^12^. *ClpB* which functions to break apart protein aggregates for proper protein refolding was up-regulated to 1.58 and 3.06 fold at 3 hour and 24 hours, respectively. This result demonstrated that with the stress of high concentration of ethanol, more ClpB protein was needed to disaggregate damaged and misfolded proteins to protect the *L. acetotolerans* cells. These results supports the expectation that a more robust protein refolding system is beneficial for cell survival under solvent stress^24^.

The dominant significantly down-regulated core genes in *L. acetotolerans* F28 treated by ethanol for both 3 hours and 24 hours were amino acid ABC transporter components encoding genes including *hisJ*, *glnQ*, *glnP* and *gltP*. The amino acid transport and metabolism pathway might be reduced to decrease energy requirements and enhance the survival system of stressed cells. This was supported by the responses of *L. plantarum* that amino acid metabolism related genes were down-regulated after 8% ethanol treatment^12^. In the VPNC cells of *L. acetotolerans*, amino acid transport and metabolism pathway were also significantly down-regulated compared to normal cells^21^, indicating that reducing “amino acid transport and metabolism” pathways might be a necessary strategy to keep carbon and nitrogen metabolism balance to survive in ethanol stressed conditions.

Fatty acid biosynthesis related genes were reported to be increased in *Lactobacilli* to protect cells against environment stresses^12, 25^. 3 genes participated in fatty acids biosynthesis were significantly up-regulated after ethanol treated for 3 hours, and 8 other related genes were significantly up-regulated at 24 hours with gene expression levels higher than those at 3 hours. Our fatty acid methyl ester analyses showed that most tested fatty acids were increased in *L. acetotolerans* F28 after exposure to ethanol, and the unsaturated fatty acid octadecenoic acid (C18:1) was significantly increased by nearly 2 fold at 24 hours. *L. acetotolerans* F28 might increase fatty acid biosynthesis to enhance cell membrane fluidity and protect the cells against high concentration of ethanol. As the hypothesis of “homeoviscous adaptation” suggested that variation of fatty-acid composition of membrane phospholipids serves in producing membranes whose lipids have a constant fluidity in the growth process^26^, we supposed that the production of a more fluid membrane in *L. acetotolerans* F28 was a compensation for the increase in “rigidity” due to the environmental ethanol stress. This behavior is consistent with the ethanol-induced fatty acid changes occurring in *Lactobacillus hilgardii* ^27^ and *Saccharomyces cerevisiae* ^28^. In the VPNC cells of *L. acetotolerans* isolated from spoilage beer, “fatty acid biosynthesis” was down-regulated compared to the normal cells. We supposed that the formation of VPNC state through ethanol and the adaption to high concentration of ethanol in *L. acetotolerans* F28 went through some different genetic mechanism. This is interesting and need further research.

According to our present results, we supposed a possible model about how *L. acetotolerans* F28 respond to 12% ethanol. When *L. acetotolerans* F28 was stimulated by ethanol, stress response and defense related genes were up-regulated rapidly in a short time to protect the cells by preventing the mis-folding and aggregation of cell proteins. HrcA related response pathway might contribute to *L. acetotolerans* F28 protection to ethanol. At the same time, the synthesis of uracil nucleotides and peptide degradation was improved, and the transport and biosynthesis of amino acids in *L. acetotolerans* F28 was decreased to deduce energy requirements and enhance the survival system of stressed cells. At a short time, fatty acids started to be accumulated after ethanol treatment to protect the cells. After a longer time exposure to 12% ethanol, more genes participated in stress response and defense were enhanced to disaggregate damaged and misfolded proteins to protect the *L. acetotolerans* cells. The transport and biosynthesis of amino acids was still decreased to adapt to the stressed condition. Fatty acids, especially unsaturated fatty acids biosynthesis were much increased to enhance cell membrane fluidity and protect the cells against high concentration of ethanol.

*L. acetotolerans* is the dominant specie from 9^th^ days during the liquor fermentation, and is also found in Japanese sake and spoiled beer. Till now, there is still limited knowledge about the function of *L. acetotolerans*. This study enlarged our knowledge of the ethanol stress tolerance mechanisms of *L. acetotolerans* in either a short or a longer time. Transcriptome analysis indicated that much higher expression levels of stress response genes and fatty acid biosynthesis related genes were necessary for *L. acetotolerans* F28 to adapt to environmental ethanol. Decreased “amino acid transport and metabolism” pathway may reduce energy requirement and maintain carbon and nitrogen metabolism balance to be survived in the stressed conditions. Elucidating the adaptive behavior of *L. acetotolerans* under ethanol stress could pave the way toward the targeted stress improvement and the study of its functions in liquor fermentation process.

## Materials and Methods

### Identification of *L. acetotolerans*

The 16 S rRNA gene of F28 was amplified by PCR using the universal primers 27 F and 1492R^29^ and sequenced. Sequence alignment and comparisons with close relatives available from GenBank were performed using EZBioCloud (http://www.ezbiocloud.net/identify) to determine an approximate phylogenetic affiliation. The phylogenetic tree were constructed with the neighbour-joining and maximum-likelihood methods using the MEGA 6 program^30^.

### Strain Culture and ethanol tolerance

*L. acetotolerans* F28 was cultured at 30 °C in MRS5 broth medium added with different concentrations of ethanol when needed. In order to see the growth of *acetotolerans* F28 at early exponential phase under ethanol stress, cells were inoculated till OD_600_ = 1. Then the culture was separated to different tubes. Different percent (v/v) of ethanol were added to each tube while water was also added as a control. The growth of *L. acetotolerans* F28 in each tube was determined by the cell density at 600 nm (OD_600_) measured by a spectrophotometer (UV-1800, SHIMADZU, Japan). All the measurements were performed in triplicate using independent cultures.

### RNA isolation and transcriptome analysis

*L. acetotolerans* F28 cells were cultured till OD_600_ = 1 and treated with 12% (v/v) ethanol or ddH_2_O (as control) for 3 and 24 hours, 3 replicates for each sample. The cells were harvested and the total RNA were extracted and purified using the Bacterial RNA kit (OMEGA^TM^) according to the protocol provided by the manufacturer. Quality control of each RNA sample was performed with Agilent 2100 Bioanalyzer. The cDNA libraries were constructed using NEBNextUltra^TM^ RNA library Prep Kit and submitted for sequencing using Illumina Hiseq 4000. The library construction and sequencing were performed by the Allwegene BioTech in Beijing (China). The processed reads were mapped to the *L. acetotolerans* NBRC 13120 genome assembly using TopHat 2.1.1^8^ with reference annotation. FPKM values for each gene and differentially expressed genes (DEGs) were analyzed with Cufflinks v2.2.1^31^. The differentially expressed genes between two samples were identified considering both fold change and q-value. The RNA-seq data discussed in this publication have been deposited in NCBI’s Gene Expression Omnibus and are accessible through GEO Series accession number GSE92732 (http://www.ncbi.nlm.nih.gov/geo/query/acc.cgi?acc=GSE92732).

### Quantitative RT-PCR (qRT-PCR) analysis

RNA-seq data were validated using real-time qRT-PCR. Kapa 2 × SYBR Green mix was used for reverse transcription of RNA to cDNA and Roche LC480 were used for amplification. Twenty four genes representing different functional categories and gene expression values based on RNA-seq data were analyzed.

### Fatty acids (FAs) extraction

*L. acetotolerans* F28 cells were cultured to OD_600_ = 1 and treated with 12% ethanol and H_2_O, independently. After 3 and 24 hours, the cells were harvested and washed with 0.9% NaCl twice. After frozen for 24 h at −40 °C, the samples were immediately freeze-dried in an freeze dryer with a condenser temperature at −96 °C and a chamber pressure of < 0.20 hpa for 48 h. 200 mg dried cells were saponified with 4 mL 2 mol/L NaOH-MeOH at 70 °C for 10 min and then methylated with 4 mL 10% H_2_SO_4_- MeOH at 70 °C for 15 min. The fatty acid methyl esters (FAME) were extracted by n-hexane, analyzed by gas-chromatograph (Agilent 7890 A) and 5975 C mass spectrometer. The MS detector was fitted to a HP-5 MS capillary column (30 m x 0.25 mm x 0.25 μm film). The carrier (helium) flow was 1 ml min^−1^. 1 µl fatty acid methyl esters was injected with a split ratio of 10:1. The injector temperature was 280 °C, the MS source temperature was 230 °C, the MS quadrupole temperature was 150 °C and the oven temperature was increased from 70 to 230 °C at 5 °C min^−1^, then 230 to 290 °C at 10 °C min^−1^ and maintained at 290 °C for 6 min. The peaks that contained ion 74 which is an indicative of a methylated lipid, were identified using the NIST database, and the area of each peak was calculated. Relative quantification of each peak was performed by the using benzoic acid as an internal standard (IS). For quantification, the area ratios of protonated molecule of a given FAME versus the IS were calculated.

### Microscopy

For scanning electron microscopy (SEM), *L. acetotolerans* F28 cells were cultured till OD_600_ = 1 and harvested after exposure to 12% (v/v) ethanol for 3 and 24 hours. Then the cells were washed with PBS and fixed with 2.5% (v/v) glutaraldehyde overnight in dark place. After rinsed with water, cells were subsequently incubated on coverslips and dehydrated by serial incubation of increasing concentration of ethanol solution from 50% to 70%, 85%, 95% and 100% ethanol. After critical point drying with carbon dioxide, the coverslips were coated with platinum-gold and were analyzed with a field emission scanning electron microscope (SU8010, Hitachi, Japan) at room temperature.

## Electronic supplementary material

Supplementary Table S1

Supplementary Table S2
