# Supplementary material for: Transcriptome responses of Lactobacillus acetotolerans F28 to a short and long term ethanol stress
Source: Sci Rep. 2017 Jun 1;7:2650. doi: 10.1038/s41598-017-02975-8 (PMC5453994; doi:10.1038/s41598-017-02975-8)
Supplement: Supplementary file 1 — Supplementary Table S1 [file 41598_2017_2975_MOESM1_ESM.docx]

**Supplementary information**

**Transcriptome responses of *Lactobacillus acetotolerans* F28 to a short and long term ethanol stress**

Xiaopan Yang^1, 2^†, Kunling Teng^1^†, Jie Zhang^1, 2^, Fangfang Wang^1, 2^, Tong Zhang^1, 2^,

Guomin Ai^1^, Peijie Han^3^, Fengyan Bai^2,3^, Jin Zhong^1, 2^*

1 State Key Laboratory of Microbial Resources, Institute of Microbiology, Chinese Academy of Sciences, Beijing 100101, People’s Republic of China

2 University of Chinese Academy of Sciences, Beijing 100101, People’s Republic of China

3 State Key Laboratory of Mycology, Institute of Microbiology, Chinese Academy of Sciences, Beijing 100101, People’s Republic of China

*Correspondence: Jin Zhong (Email: [zhongj@im.ac.cn](mailto:zhongj@im.ac.cn)), State Key Laboratory of Microbial Resource, Institute of Microbiology, Chinese Academy of Sciences. NO. 1 Beichen West Road, Chaoyang District, Beijing 100101, RP China. Fax: +8610 64807401.

†These authors have contributed equally to this work and should be considered co-first authors.

**Supplementary Table S1** Significantly differentially expressed genes *L. acetotolerans* F28 treated with ethanol for 3 hours

| Gene ID | Gene | Protein encoded | log2_fold_change |
| --- | --- | --- | --- |
| LBAT_0594 | *pyrR* | Uracil phosphoribosyltransferase | 3.20445 |
| LBAT_0595 | *pyrB* | Aspartate carbamoyltransferase catalytic subunit | 6.17615 |
| LBAT_0596 | *pyrC* | Dihydroorotase | 4.8697 |
| LBAT_0597 | *carA* | Carbamoyl phosphate synthase small subunit | 3.75128 |
| LBAT_0598 | *carB* | Truncated carbamoyl phosphate synthase large subunit | 2.79192 |
| LBAT_1269 | *pyrR* | Uracil phosphoribosyltransferase | 2.98867 |
| LBAT_0230 | *hsp20* | Heat shock protein | 2.51861 |
| LBAT_1270 | *uraA* | Uracil/xanthine transporter | 2.49444 |
| LBAT_0456 | *groEL* | Chaperone GroEL | 2.26589 |
| LBAT_0711 | *hrcA* | Heat-inducible transcription repressor | 1.91573 |
| LBAT_0713 | *dnaK* | Molecular chaperone DnaK | 1.87137 |
| LBAT_1215 | *clpE* | Clp protease ClpE | 1.80356 |
| LBAT_1353 | *adhE* | Aldehyde-alcohol dehydrogenase | 1.79571 |
| LBAT_0455 | *groES* | Chaperone GroES | 1.77969 |
| LBAT_0262 | *—* | Transcriptional regulator | 1.7511 |
| LBAT_0712 | *grpE* | Heat shock protein GrpE | 1.73331 |
| LBAT_0819 | *citT* | Anion permease | 1.64382 |
| LBAT_0164 | *nagA* | N-acetylglucosamine-6-phosphate deacetylase | 1.61642 |
| LBAT_0174 | *—* | Hypothetical protein | 1.60635 |
| LBAT_1266 | *araJ* | MFS transporter | 1.5445 |
| LBAT_0714 | *dnaJ* | Chaperone protein DnaJ | 1.53906 |
| LBAT_0412 | *—* | Conserved hypothetical protein | 1.45488 |
| LBAT_0999 | *—* | Conserved hypothetical protein | 1.4093 |
| LBAT_1471 | *—* | Nucleoside triphosphate（NTP） hydrolase | 1.38512 |
| LBAT_0588 | *soxR* | MerR family transcriptional regulator | 1.3557 |
| LBAT_0133 | *mpg* | 3-methyladenine DNA glycosylase | 1.35085 |
| LBAT_1494 | *—* | Transcriptional regulator | 1.32052 |
| LBAT_0796 | *sdhA* | Fumarate reductase flavoprotein subunit | 1.31394 |
| LBAT_0818 | *ubiE* | Dimethylmenaquinone methyltransferase | 1.29069 |
| LBAT_1391 | *hisM* | Amino acid ABC transporter permease component | 1.24952 |
| LBAT_0095 | *hxlR* | Transcriptional regulator | 1.24149 |
| LBAT_1440 | *—* | Hypothetical protein | 1.23403 |
| LBAT_0616 | [*fabZ*](http://www.wikigenes.org/e/gene/e/1795859.html) | (3R)-hydroxymyristoyl-[acyl carrier protein | 1.22865 |
| LBAT_1000 | *rnj* | Ribonuclease J | 1.19991 |
| LBAT_1527 | *natA* | Na+ ABC transporter ATP-binding protein | 1.18368 |
| LBAT_0132 | *—* | Amino acid ABC transporter substrate-binding protein | 1.17326 |
| LBAT_0060 | *dkgA* | 2,5-diketo-D-gluconic acid reductase | 1.17095 |
| LBAT_0096 | *pfpI1* | PfpI family intracellular protease | 1.15116 |
| LBAT_1496 | *—* | Hypothetical protein | 1.15018 |
| LBAT_0321 | *yafJ* | Glutamine amidotransferase | 1.13576 |
| LBAT_0307 | *pepD* | Dipeptidase | 1.12658 |
| LBAT_0690 | *plsC* | 1-acyl-sn-glycerol-3-phosphate acyltransferase | 1.10363 |
| LBAT_1441 | *ftsE* | ABC transporter ATP-binding component | 1.07825 |
| LBAT_0219 | *galM* | Aldose epimerase | 1.06561 |
| LBAT_1478 | *relB* | Hypothetical protein | 1.058 |
| LBAT_0599 | *carB* | Truncated carbamoyl phosphate synthase large subunit | 1.05502 |
| LBAT_1121 | *mtlA* | PTS system mannitol-specific IIBC components | 1.04863 |
| LBAT_1568 | *—* | Hypothetical protein | 1.01912 |
| LBAT_0868 | *—* | Hypothetical protein | 1.01827 |
| LBAT_0313 | *uraA* | Uric acid permease PucJ | 1.0134 |
| LBAT_1497 | *—* | [Hypothetical protein](https://blast.ncbi.nlm.nih.gov/Blast.cgi#alnHdr_983173026) | 1.01117 |
| LBAT_0316 | *—* | Hypothetical protein | 1.0072 |
| LBAT_1499 | *—* | Hypothetical protein | Inf |
| LBAT_0319 | *—* | Hypothetical protein | Inf |
| LBAT_1449 | *mdlB* | ABC transporter ATP-binding component | -1.00488 |
| LBAT_0907 | *ppx* | Manganese-dependent inorganic pyrophosphatase | -1.0137 |
| LBAT_0054 | *—* | Hypothetical protein | -1.02238 |
| LBAT_1123 | [*glmS*](http://www.wikigenes.org/e/gene/e/3994403.html) | Glutamine-fructose-6-phosphate transaminase | -1.03413 |
| LBAT_0642 | *—* | Hypothetical protein | -1.05993 |
| LBAT_0718 | [*rimI*](http://www.wikigenes.org/e/gene/e/1462488.html) | GNAT family acetyltransferase | -1.06482 |
| LBAT_0556 | *phoR* | Two-component sensor kinase | -1.07269 |
| LBAT_0103 | *rlmH* | rRNA large subunit methyltransferase | -1.07714 |
| LBAT_1548 | *potE* | Amino acid permease | -1.07977 |
| LBAT_0185 | *kup* | Potassium transporter Kup | -1.08146 |
| LBAT_0175 | *—* | Transcriptional regulator | -1.0819 |
| LBAT_0196 | *cwlA* | Amidase | -1.10191 |
| LBAT_1640 | *dnk* | Deoxyadenosine kinase | -1.16607 |
| LBAT_0373 | *rpmD* | 50S ribosomal protein L30 | -1.22615 |
| LBAT_0053 | *cobA* | Cobalamin adenosyltransferase | -1.22637 |
| LBAT_0937 | *—* | Hypothetical protein | -1.23024 |
| LBAT_0165 | [*dnph1*](http://www.wikigenes.org/e/gene/e/171047.html) | Nucleoside 2-deoxyribosyltransferase | -1.25444 |
| LBAT_1573 | *eno* | Truncated enolase | -1.27345 |
| LBAT_1111 | *—* | NAD(FAD)-dependent dehydrogenase | -1.3111 |
| LBAT_1641 | *dck* | Deoxyguanosine kinase | -1.38333 |
| LBAT_0264 | *murQ* | N-acetylmuramic acid 6-phosphate etherase | -1.47895 |
| LBAT_0193 | *—* | Hypothetical protein | -1.60071 |
| LBAT_0325 | *glnP* | Polar amino acid ABC transporter permease component | -1.71422 |
| LBAT_0326 | *glnP* | Amino acid ABC transporter permease component | -1.82082 |
| LBAT_0327 | *glnQ* | Amino acid ABC transporter ATP-binding component | -2.35099 |
| LBAT_0869 | *gltP* | Sodium/dicarboxylate symporter | -2.3925 |
| LBAT_0328 | *hisJ* | Amino acid ABC transporter substrate binding component | -2.61036 |

Grey：Genes of downregulation

—：Unkown
