# Supplementary material for: Transcriptome responses of Lactobacillus acetotolerans F28 to a short and long term ethanol stress
Source: Sci Rep. 2017 Jun 1;7:2650. doi: 10.1038/s41598-017-02975-8 (PMC5453994; doi:10.1038/s41598-017-02975-8)
Supplement: Supplementary file 2 — Supplementary Table S2 [file 41598_2017_2975_MOESM2_ESM.docx]

**Supplementary information**

**Transcriptome responses of *Lactobacillus acetotolerans* F28 to a short and long term ethanol stress**

Xiaopan Yang^1, 2^†, Kunling Teng^1^†, Jie Zhang^1, 2^, Fangfang Wang^1, 2^, Tong Zhang^1, 2^,

Guomin Ai^1^, Peijie Han^3^, Fengyan Bai^2,3^, Jin Zhong^1, 2^*

1 State Key Laboratory of Microbial Resources, Institute of Microbiology, Chinese Academy of Sciences, Beijing 100101, People’s Republic of China

2 University of Chinese Academy of Sciences, Beijing 100101, People’s Republic of China

3 State Key Laboratory of Mycology, Institute of Microbiology, Chinese Academy of Sciences, Beijing 100101, People’s Republic of China

*Correspondence: Jin Zhong (Email: [zhongj@im.ac.cn](mailto:zhongj@im.ac.cn)), State Key Laboratory of Microbial Resource, Institute of Microbiology, Chinese Academy of Sciences. NO. 1 Beichen West Road, Chaoyang District, Beijing 100101, RP China. Fax: +8610 64807401.

†These authors have contributed equally to this work and should be considered co-first authors.

**Supplementary Table S2** Significantly differentially expressed genes *L. acetotolerans* F28 treated with ethanol for 24 hours

| Gene ID | Gene | Protein encoded | log2_fold_change |
| --- | --- | --- | --- |
| LBAT_1545 | *gmpR2* | Guanosine monophosphate reductase | 2.42685 |
| LBAT_1280 | *—* | Hypothetical protein | 1.95724 |
| LBAT_0622 | *birA* | Biotin-[acetyl-CoA-carboxylase] ligase | 1.94815 |
| LBAT_1353 | *adhE* | Aldehyde-alcohol dehydrogenase | 1.87131 |
| LBAT_0089 | *plsC* | Acyl-phosphate glycerol 3-phosphate acyltransferase | 1.75659 |
| LBAT_1571 | *—* | Hypothetical protein | 1.71733 |
| LBAT_1532 | *thiM* | Hydroxyethylthiazole kinase | 1.68943 |
| LBAT_1574 | *—* | Hypothetical protein | 1.67093 |
| LBAT_1316 | *mgtA* | Calcium-translocating P-type ATPase, PMCA-type | 1.64904 |
| LBAT_1554 | *clpB* | ATP-dependent Clp protease ATP-binding subunit | 1.61198 |
| LBAT_1379 | *—* | Hypothetical protein | 1.58072 |
| LBAT_0191 | *—* | Hypothetical protein | 1.56719 |
| LBAT_0075 | *dnaA* | DNA replication initiation protein | 1.55853 |
| LBAT_1473 | *pgam* | Phosphoglycerate mutase | 1.55639 |
| LBAT_0230 | *hsp20* | Heat shock protein | 1.54241 |
| LBAT_0677 | *pnp* | Phosphorylase | 1.54011 |
| LBAT_1464 | *—* | TetR family transcriptional regulator | 1.53667 |
| LBAT_1279 | *rimI* | Acetyltransferase | 1.53223 |
| LBAT_1333 | *rimI* | GCN5 family acetyltransferase | 1.47393 |
| LBAT_1104 | *ftsE* | Macrolide ABC transporter ATP-binding protein | 1.42537 |
| LBAT_0605 | *—* | Hypothetical protein | 1.41737 |
| LBAT_1092 | *—* | DNA methylase | 1.35798 |
| LBAT_0064 | *bglF* | PTS system trehalose-specific IIABC components | 1.34763 |
| LBAT_1415 | *gmk* | Guanylate kinase | 1.31377 |
| LBAT_0078 | *sdhA* | Fumarate reductase flavoprotein subunit | 1.28994 |
| LBAT_1544 | *purA* | Adenylosuccinate synthase | 1.28135 |
| LBAT_0776 | *—* | Hypothetical protein | 1.28021 |
| LBAT_1105 | *skp* | ABC transporter permease component | 1.25591 |
| LBAT_0067 | *ldhA* | D-lactate dehydrogenase | 1.24791 |
| LBAT_1107 | *ftsX* | ABC transporter ATP-binding component | 1.23984 |
| LBAT_1352 | *rimL* | Acetyltransferase | 1.23955 |
| LBAT_1543 | *purB* | Adenylosuccinate lyase | 1.22445 |
| LBAT_0588 | *soxR* | MerR family transcriptional regulator | 1.21159 |
| LBAT_0093 | *—* | Hypothetical protein | 1.20787 |
| LBAT_0800 | *copA* | Copper-translocating P-type ATPase | 1.2017 |
| LBAT_1344 | *yadH* | Putative ABC transporter permease component | 1.19193 |
| LBAT_1525 | *thiD* | Phosphomethylpyrimidine kinase | 1.1891 |
| LBAT_0755 | *deoC* | Deoxyribose-phosphate aldolase | 1.18216 |
| LBAT_0628 | *aes* | Truncated lipase | 1.16727 |
| LBAT_0321 | *yafJ* | Glutamine amidotransferase | 1.16209 |
| LBAT_1478 | *relB* | Hypothetical protein | 1.14112 |
| LBAT_0068 | *aphA* | Aminoglycoside phosphotransferase | 1.13719 |
| LBAT_0095 | *hxlR* | Transcriptional regulator | 1.10428 |
| LBAT_0176 | *—* | XRE family transcriptional regulator | 1.09343 |
| LBAT_0088 | *rfaJ* | Glycosyltransferase | 1.09297 |
| LBAT_0764 | *rfaJ* | Glycosyltransferase | 1.08677 |
| LBAT_0412 | *—* | Conserved hypothetical protein | 1.08545 |
| LBAT_0133 | *mpg* | 3-methyladenine DNA glycosylase | 1.07526 |
| LBAT_0146 | *wcaA* | Glycosyl transferase | 1.07263 |
| LBAT_1556 | *—* | Acyltransferase | 1.06984 |
| LBAT_0627 | *aes* | Truncated lipase | 1.06685 |
| LBAT_1471 | *—* | Nucleoside triphosphate（NTP） hydrolase | 1.06174 |
| LBAT_0809 | *dapE* | Succinyl-diaminopimelate desuccinylase | 1.05996 |
| LBAT_1275 | *—* | AbiH Superfamily | 1.05905 |
| LBAT_1377 | *—* | Hypothetical protein | 1.05184 |
| LBAT_1566 | *—* | Hypothetical protein | 1.04965 |
| LBAT_0162 | *—* | Hypothetical protein | 1.04947 |
| LBAT_0711 | *hrcA* | Heat-inducible transcription repressor | 1.04283 |
| LBAT_0015 | *—* | Esterase | 1.03826 |
| LBAT_1274 | *—* | Hypothetical protein | 1.03306 |
| LBAT_0675 | *—* | Hypothetical protein | 1.03094 |
| LBAT_1557 | *celB* | PTS system cellobiose-specific IIC component | 1.02786 |
| LBAT_1627 | *trxA* | Thiol reductase thioredoxin | 1.02004 |
| LBAT_1467 | *relB* | RelB | 1.01976 |
| LBAT_0126 | *—* | Hypothetical protein | 1.00433 |
| LBAT_1277 | *—* | Hypothetical protein | Inf |
| LBAT_0901 | *opuABC* | Glycine/betaine ABC transporter permease | -1.00301 |
| LBAT_1472 | *pgam* | Phosphoglycerate mutase | -1.00707 |
| LBAT_1257 | *glnP* | Glutamine ABC transporter permease component | -1.0282 |
| LBAT_1283 | *tpx* | 2-Cys peroxiredoxin | -1.0308 |
| LBAT_1393 | *—* | Hypothetical protein | -1.03301 |
| LBAT_0931 | *yfgC* | Peptide-binding protein | -1.03352 |
| LBAT_0603 | *arsC* | Arsenate reductase | -1.03666 |
| LBAT_1229 | *paaJ* | Truncated acetyl-CoA acetyltransferase | -1.04342 |
| LBAT_0080 | *sdhA* | Fumarate reductase flavoprotein subunit | -1.04674 |
| LBAT_1148 | *hdeD* | Hypothetical protein | -1.05559 |
| LBAT_0347 | *clpC* | ATP-dependent Clp protease ATP-binding protein ClpC | -1.05569 |
| LBAT_0195 | *acm* | N-acetylmuramidase | -1.06058 |
| LBAT_0556 | *phoR* | Two-component sensor kinase | -1.06735 |
| LBAT_0751 | *metQ* | ABC transporter ATP-binding component | -1.07207 |
| LBAT_0585 | *—* | Hypothetical protein | -1.08385 |
| LBAT_0683 | *lexA* | LexA repressor | -1.1191 |
| LBAT_0196 | *cwlA* | Amidase | -1.12182 |
| LBAT_1373 | *potA* | Sugar ABC transporter ATP-binding component | -1.13174 |
| LBAT_0880 | *nth* | Endonuclease III | -1.14208 |
| LBAT_1623 | *—* | Hypothetical protein | -1.14723 |
| LBAT_0333 | *alr* | Alanine racemase | -1.14832 |
| LBAT_1049 | *ftsW* | Cell division membrane protein | -1.1763 |
| LBAT_1650 | *pox* | Pyruvate oxidase | -1.18216 |
| LBAT_1371 | *ugpE* | Sugar ABC transporter permease component | -1.18597 |
| LBAT_0933 | *der* | Ribosome biogenesis GTPase Der | -1.20208 |
| LBAT_0836 | *yitT* | Membrane protein | -1.21817 |
| LBAT_1228 | *paaJ* | Truncated acetyl-CoA acetyltransferase | -1.24707 |
| LBAT_1372 | *potC* | Sugar ABC transporter permease component | -1.25218 |
| LBAT_0121 | *—* | Hypothetical protein | -1.25696 |
| LBAT_0085 | *cyt-b5* | Hypothetical protein cytochrome b5 superfamily | -1.26463 |
| LBAT_0348 | *rpoB* | DNA-directed RNA polymerase beta subunit | -1.27492 |
| LBAT_0527 | *yhaM* | 3'-5' exonuclease | -1.29326 |
| LBAT_0827 | *ybaK* | Aminoacyl-tRNA deacylase | -1.30379 |
| LBAT_0937 | *—* | Hypothetical protein | -1.30454 |
| LBAT_1227 | *hmg1* | Hydroxymethylglutaryl-CoA reductase | -1.36862 |
| LBAT_1147 | *rapZ* | RNase adaptor protein RapZ | -1.39126 |
| LBAT_0131 | *glnQ* | Amino acid ABC transporter ATP-binding component | -1.41149 |
| LBAT_0753 | *metP* | Methionine ABC transporter permease | -1.42485 |
| LBAT_0838 | *rpsU* | 30S ribosomal protein S21 | -1.45124 |
| LBAT_0869 | *gltP* | Sodium/dicarboxylate symporter | -1.46471 |
| LBAT_0349 | *rpoC* | DNA-directed RNA polymerase beta' subunit | -1.50151 |
| LBAT_1370 | *—* | MBL fold metallo-hydrolase | -1.52436 |
| LBAT_1146 | *yvcK* | Hypothetical protein | -1.55662 |
| LBAT_1369 | *ugpB* | Glycerol-3-phosphate ABC transporter substrate binding component | -1.56036 |
| LBAT_0835 | *msrA* | Methionine sulfoxide reductase A | -1.56904 |
| LBAT_0258 | *ampC* | Penicillin-binding protein | -1.58595 |
| LBAT_0752 | *metN* | Methionine ABC transporter ATP-binding protein | -1.59324 |
| LBAT_0512 | *—* | Alpha/beta hydrolase | -1.63497 |
| LBAT_0096 | *pfpI1* | PfpI family intracellular protease /glutamine amidotransfera | -1.63576 |
| LBAT_1145 | *whiA* | Sporulation regulator/DNA-binding protein WhiA | -1.64333 |
| LBAT_0834 | *msrB* | Methionine sulfoxide reductase B | -1.66141 |
| LBAT_1226 | *hmgcs2* | Hydroxymethylglutaryl-CoA synthase | -1.67955 |
| LBAT_1421 | *ftsP* | Truncated multicopper oxidase | -1.69468 |
| LBAT_0908 | *lysR* | ArsR family transcriptional regulator | -1.8292 |
| LBAT_1255 | *dps* | DNA starvation/stationary phase protection protein | -1.86427 |
| LBAT_0847 | *rpoD* | RNA polymerase sigma factor RpoD | -1.96128 |
| LBAT_1342 | *—* | NADH peroxidase | -1.9667 |
| LBAT_1254 | *—* | Hypothetical protein | -1.9722 |
| LBAT_1420 | *—* | Truncated multicopper oxidase | -2.08819 |
| LBAT_1111 | *—* | NAD(FAD)-dependent dehydrogenase | -2.21482 |
| LBAT_0325 | *glnP* | Polar amino acid ABC transporter permease component | -2.33701 |
| LBAT_0326 | *glnP* | Amino acid ABC transporter permease component | -2.45301 |
| LBAT_0328 | *hisJ* | Amino acid ABC transporter substrate binding component | -2.47179 |
| LBAT_0327 | *glnQ* | Amino acid ABC transporter ATP-binding component | -2.54763 |

Grey：Genes of downregulation

—：Unkown
